# Use of graded responsibility and common entrustment considerations among United States emergency medicine residency programs

**DOI:** 10.3352/jeehp.2020.17.11

**Published:** 2020-04-20

**Authors:** Jason Lai, Benjamin Holden Schnapp, David Simon Tillman, Mary Westergaard, Jamie Hess, Aaron Kraut

**Affiliations:** BerbeeWalsh Department of Emergency Medicine, University of Wisconsin School of Medicine and Public Health, Madison, WI, USA; Hallym University, Korea

**Keywords:** Cross-sectional studies, Emergency medicine, Internship and residency, Medical education, United States

## Abstract

**Purpose:**

The Accreditation Council for Graduate Medical Education (ACGME) requires all residency programs to provide increasing autonomy as residents progress through training, known as graded responsibility. However, there is little guidance on how to implement graded responsibility in practice and a paucity of literature on how it is currently implemented in emergency medicine (EM). We sought to determine how EM residency programs apply graded responsibility across a variety of activities and to identify which considerations are important in affording additional responsibilities to trainees.

**Methods:**

We conducted a cross-sectional study of EM residency programs using a 23-question survey that was distributed by email to 162 ACGME-accredited EM program directors. Seven different domains of practice were queried.

**Results:**

We received 91 responses (56.2% response rate) to the survey. Among all domains of practice except for managing critically ill medical patients, the use of graded responsibility exceeded 50% of surveyed programs. When graded responsibility was applied, post-graduate year (PGY) level was ranked an “extremely important” or “very important” consideration between 80.9% and 100.0% of the time.

**Conclusion:**

The majority of EM residency programs are implementing graded responsibility within most domains of practice. When decisions are made surrounding graded responsibility, programs still rely heavily on the time-based model of PGY level to determine advancement.

## Introduction

### Background/rationale

It is relatively intuitive that a resident physician nearing graduation should be entrusted with more responsibility than a first-year resident at the beginning of training. Indeed, the Accreditation Council for Graduate Medical Education (ACGME) mandates that all programs allow trainees to take on steadily more autonomy as they progress through residency training [1]. This principle of entrusting more advanced trainees with an increased level of authority is known as graded responsibility. Despite its mandate, the ACGME provides little specific guidance in its written standards for how this ideal should be executed in practice [1]. As a result, many residency training programs have traditionally awarded progressive responsibility based solely upon years of experience. With his landmark paper published in 2005, Ten Cate [2] introduced the concept of entrustable professional activities (EPAs), which represent discrete tasks that medical trainees must master in order to be deemed competent to practice independently by supervising clinicians. The goal is a new era of competency-based medical education (CBME), where trainees may theoretically be granted increasing levels of responsibility for independent practice based upon objective assessments. While this method of awarding graded responsibility holds significant promise, there is a relative paucity of guidance in the literature about if and how CBME is being practically implemented.

Previous work has attempted to elucidate the ideal system for implementing graded responsibility within residency training through panel discussions and iterative theme generation [3]. Although structured discussions yielded broad concepts and ideals that could promote graded responsibility, the panel was not instructed to enumerate specific practices that would exemplify the consensus themes that it put forward, and its findings and conclusions are not specific to emergency medicine (EM) residency programs. The literature does contain at least one example of a successful competency-based supervising experience for senior EM residents implemented at a single institution [4]. Other medical specialties and Canadian EM programs have also begun to implement CBME to assign responsibility to residents based on demonstrated abilities, but this paradigm has not yet been widely adopted and no common set of EPAs has been defined for EM [5,6]. Overall, there is a paucity of literature describing the current landscape of how graded responsibility is implemented among EM residency programs across the United States.

### Objectives

The goal of this study is to explore the ways in which graded responsibility concepts are currently utilized by EM residency programs in the United States within their curriculum and clinical environment. Understanding the current methods of implementation of graded responsibility will enable the establishment of best practices in the future. We hypothesized that more than half of EM residency programs are employing graded responsibility within each surveyed domain. We also hypothesized that post-graduate year (PGY) level, a time-based gradation, would be the most common strategy used to entrust greater levels of responsibility to trainees.

## Methods

### Ethics Statement

This study was exempt from Institutional Review Board review at the University of Wisconsin School of Medicine and Public Health.

### Study design

We conducted a survey-based cross-sectional study of EM residency programs in order to elucidate current program practices regarding graded responsibility.

### Measurement

A 23-question web-based survey was created to assess how ACGME-accredited EM residency programs implement graded responsibility among trainees across multiple domains of practice (Supplement 1). Literature review did not reveal standard domains for graded responsibility. Thus, we convened an expert panel consisting of 3 board-certified emergency physicians from the same academic department of EM. All panel members held departmental leadership roles in resident and medical student education. The expert panel established domains of practice to study and reached consensus using nominal group technique, a tool that has been used successfully in other contexts within higher education [7]. We pared down the initial list of domains to those we deemed would most likely capture the variety of methods of graded responsibility. Without robust data, expert consensus was used to determine the following 7 domains of clinical practice to query: intubating trauma patients, managing critically ill trauma patients, managing critically ill medical patients, acting as physician-in-triage, supervising medical students, supervising junior residents, and moonlighting. For each of the 7 domains, respondents were presented with 1 multiple-choice question, 1 matrix consisting of a 5-point Likert scale across 7 categories, and 1 free-text entry field. Two additional questions were used for program demographic and identification purposes (Supplement 2). Survey questions were generated with the assistance of the University of Wisconsin Survey Center, and then further assessed for response process validity by assistant program directors who had not generated the questions prior to distribution.

### Setting

The survey was generated in Qualtrics (Provo, UT, USA) and distributed to a total of 162 ACGME-accredited EM residency programs between April 2018 and October 2018. Survey links were distributed to residency program directors via email. Names and email addresses were obtained from the Council of Emergency Medicine Residency Directors mailing list and programs’ public websites. A total of up to 3 email reminders were sent to potential respondents prior to the close of the survey. Responses were screened by the primary study author (J.L.) to ensure only 1 response was received from each individual residency program. In the event of duplicate entries, only the most recent response was recorded.

### Statistical methods

Data were stored on a secure Qualtrics account, and were tabulated and analyzed using Microsoft Excel (Microsoft Corp., Redmond, WA, USA). We used descriptive statistics to analyze our data set (Dataset 1).

## Results

### 1. Included responses

**Fig. 1.**
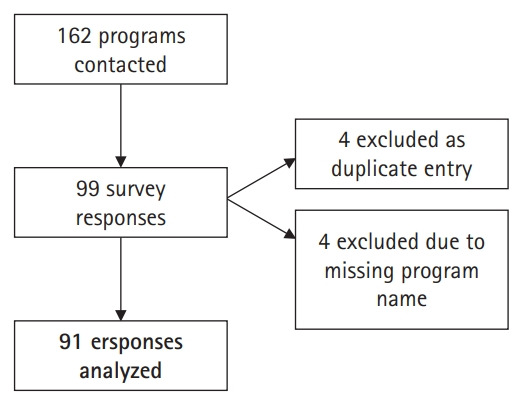
Survey responses and excluded entries.

We received a total of 99 responses to the survey. Four programs were found to have submitted 2 responses each. For each of these duplicate pairs, the most recent entry was recorded and the earlier entry excluded. Another 4 responses were excluded because the program name was missing and therefore could not be screened as a potentially duplicate entry. Ninety-one individual programs’ responses (56.2% response rate) were recorded and analyzed (Fig. 1).

### 2. Intubating trauma patients

Forty-nine residency programs (53.8%) reported that only some of their residents were allowed to intubate trauma patients, while the remaining 42 (46.1%) reported that all of their residents were allowed to intubate trauma patients (Table 1). Of those programs who only allowed some of their residents to intubate trauma patients, 38 (80.9%) rated PGY level to be an “extremely important” or “very important” criterion in determining which residents were allowed to intubate trauma patients. Completion of a certain rotation and direct observation of a previous intubation were each rated either extremely or very important by 25 respondents (53.2%). All other surveyed criteria (Clinical Competency Committee [CCC] recommendations, faculty evaluations, simulation, and milestone assessment) were each rated extremely or very important by 12 programs (25.5%) or fewer (Table 2).

3. Managing critically ill trauma patients

Fifty-three programs (59.6%) reported that only some of their residents were allowed to manage critically ill trauma patients, with the remaining 36 (40.4%) stating that all of their residents were allowed to manage critically ill trauma patients. Among programs that only allowed some of their residents to manage critically ill trauma patients, 47 (94.0%) responded that PGY level is an “extremely” or “very” important consideration in deciding which residents were allowed to perform this task. Twenty-three (46.9%) noted completion of a certain rotation to be “extremely” or “very” important. All other surveyed criteria were each rated extremely or very important by 17 programs (34.0%) or fewer.

### 4. Managing critically ill medical patients

Twenty-six programs (29.9%) reported only some of their residents being allowed to manage critically ill medical patients, and the remaining 61 (70.1%) stated that all of their residents were allowed to manage critically ill medical patients. Among programs that only allowed some residents to manage critically ill medical patients, 22 (88.0%) rated PGY level as an “extremely” or “very” important method in deciding which residents were allowed to perform this task. All other surveyed criteria were each rated extremely or very important by 9 programs (36.0%) or fewer.

### 5. Acting as physician-in-triage

Physician-in-triage is an arrangement in which an emergency physician takes the place of the traditional triage nurse in triaging newly arrived emergency department patients, which also allows for more complex physician orders to be entered earlier in the course of a patient’s stay. Forty-seven respondents (55.3%) stated that their institution does not implement physician-in-triage. Of the remaining 38 programs (44.7%) who do utilize physician-in-triage, 19 (50.0%) of these reported that only some residents were allowed to serve in the physician-in-triage role. Five (13.2%) reported all residents being allowed to serve as a physician-in-triage, and 14 (36.8%) stated that none of their residents were allowed to act as physician-in-triage. All programs that allowed only some residents to act as physician-in-triage rated PGY level as an “extremely” or “very” important criterion in determining which residents are allowed to serve in this role. All other surveyed criteria were each rated extremely or very important by 6 (31.6%) or fewer programs.

### 6. Supervising medical students

Three programs (3.5%) reported that their institution does not have medical students. Of the remaining 82 programs (96.5%), 56 (68.3%) reported only some of their residents were allowed to supervise medical students. Twenty-six (31.7%) reported all of their residents were allowed to supervise medical students, and no programs stated that none of their residents were allowed to supervise medical students. Of the programs that only allowed some residents to supervise medical students, 52 (92.9%) reported PGY level to be an “extremely important” or “very important” criterion in deciding which residents were allowed to supervise students. All other surveyed criteria were each felt to be extremely or very important by 17 programs (30.9%) or fewer.

### 7. Supervising junior residents

Thirty-six programs (50.0%) reported only allowing some of their residents to supervise junior residents. Twenty-six programs (36.1%) allowed all residents to supervise junior residents, and 10 (13.9%) allowed none of their residents to do so. All programs that allowed only some residents to supervise junior residents reported PGY level being an “extremely important” or “very important” criterion in deciding which residents were allowed to assume this responsibility. CCC recommendations and faculty evaluations were reported to be either extremely or very important by 20 programs (55.6%) and 15 programs (41.7%), respectively.

### 8. Moonlighting

Seventy-seven (90.6%) respondents reported that only some of their residents were allowed to moonlight. Two (2.4%) stated that all of their residents were allowed to, and 6 programs (7.1%) didn’t allow any of their residents to moonlight. All programs that allowed only some residents to moonlight rated PGY level as either an “extremely important” or “very important” consideration in determining which residents were allowed to moonlight. CCC recommendations were rated extremely or very important by 55 programs (71.4%). Forty-five programs (58.4%) rated faculty evaluations as extremely or very important. Milestone assessment was rated extremely or very important by 36 programs (47.4%). All other surveyed criteria were reported as extremely or very important by 25 programs (32.9%) or fewer.

## Discussion

### Key results

In line with our hypothesis, our survey responses demonstrate that the majority of EM residency programs are implementing graded responsibility for most surveyed domains of practice. With the exception of managing critically ill medical patients, every other surveyed domain had greater than 50% of programs stating that only some of their residents were allowed to perform that task, implying that graded responsibility was being applied to that domain. This finding is consistent with previous research demonstrating that graded responsibility is commonly found in other sectors of graduate medical education outside of EM [8]. Also, in line with our hypothesis, PGY level was the most highly valued criterion in determining whether a resident was entrusted with greater responsibility across every surveyed domain.

### Interpretation

PGY level appears to consistently be the most important consideration utilized by residency leadership to entrust additional responsibility to residents across all of the investigated clinical and educational domains. However, faculty input, in the form of both individual evaluations and recommendations from the CCC, was also cited as an important consideration for a significant minority of programs. Finally, “observation of having performed task previously” was most valued when determining graded responsibility for the intubation of trauma patients, suggesting that some procedural competencies may lend themselves better to assessment via direct observation or completion of a focused rotation. However, workplace-based assessment models do exist such as the mini-Clinical Evaluation Exercise and Clinical Work Sampling which may allow faculty to reliably assess more abstract domains such as ability to act as physician in triage [9]. Overall, this suggests that some programs may be taking into account individual differences in skills progression during residency, making progress toward the truly individualized educational experience promised by a CBME model [10]. However, our data shows that this experience is far from universal. Our study also did not investigate the form that this data takes, such as faculty gestalt or discrete rating scales, which could be an avenue for future research; a true competency-based model would expect residency programs to collect a significant number of concrete evaluations in order to make a valid entrustment decision [11].

Across all surveyed domains of clinical practice, there was a notable minority of programs not using graded responsibility according to the results of our survey. The management of critically ill medical patients was most frequently allocated to residents of any level: 61 programs (70.1%) allowed all residents this responsibility. Conversely, moonlighting was the most restrictive domain of practice, with only 2 (2.4%) surveyed programs allowing all residents access. Six (7.1%) surveyed residency programs do not allow moonlighting at all, consistent with previously reported literature [12]. Perhaps because moonlighting most closely resembles the full duties of an emergency physician who has completed the entirety of residency, our findings suggest that multiple considerations are included in the decision to allow a resident to moonlight, more so than for other surveyed responsibilities. Specifically, PGY year, CCC recommendations, and faculty evaluations are extremely or very important for more than half of programs that allow moonlighting, and 36 programs (47.4%) also consider milestones.

### Limitations

One limitation of our study is that we used expert consensus to determine the domains of graded responsibility that we assessed with our survey instrument. There could potentially be other important graded responsibility opportunities not included in the final version of our survey. Also, our survey instrument design, which asks the importance of individual considerations independently, may fail to fully capture the complexity of graded responsibility assignments. Additionally, the nature of graded responsibility itself is often a spectrum of decreasing oversight rather than a binary decision about whether a learner is allowed to perform a task or not, a nuance that may not be fully captured by the questions in our survey instrument. For example, situations such as a junior resident co-managing a critically ill medical patient with a more senior physician may be difficult to accurately categorize, and therefore terms used in the survey instrument such as “managing” and “supervising” may be interpreted differently by different respondents. Variable interpretation of a survey question also arises when examining responses regarding supervising junior residents, as a proportion of the 26 respondents (36.1%) who allowed all residents to supervise junior residents may have presumed the choice referred to more senior residents and not those of the same level. Finally, our data are subject to potential biases such as response bias [13] and sampling bias [14] inherent to survey-based investigation.

### Conclusion

Overall, our study suggests that EM residency programs still rely heavily on a time-based learning model when applying graded responsibility, and that broad implementation of competency-based educational models does not yet appear to be the norm within the United States. CCCs and individual EM faculty also currently have significant influence on the progression of residents through certain graded responsibilities. While the ACGME officially launched “milestones” in EM in 2013 [15], the transition to outcomes-based medical education remains incomplete at best. With EPAs on the horizon as the next step in competency-oriented education, the results of this survey serve as a reminder that time-based modalities still drive the gradation of responsibility across most domains. However, competency-based graded responsibility appears to have traction in decisions regarding trauma intubations, trauma critical care, and moonlighting. Further research is needed to investigate program characteristics that may be associated with implementation of CBME, existing barriers to implementation, as well as potential avenues for more widespread adoption.

## Figures and Tables

**Table 1.** Utilization of graded responsibility among surveyed emergency medicine residency programs

Domain of practice	All residents allowed to	Only some residents allowed to	No residents allowed to	Not applicable
Intubating trauma patients	42 (46.2)	49 (53.8)	0	-
Managing critically ill trauma patients	36 (40.4)	53 (59.6)	0	-
Managing critically ill medical patients	61 (70.1)	26 (29.9)	0	-
Acting as physician-in-triage	5 (13.2)^a)^	19 (50.0)^a)^	14 (36.8)^a)^	47
Supervising medical students	26 (31.7)^a)^	56 (68.3)^a)^	0	3
Supervising junior residents	26 (36.1)^a)^	36 (50.0)^a)^	10 (13.9)^a)^	7
Moonlighting	2 (2.4)	77 (90.6)	6 (7.1)	-

Values are presented as number (%).

a) Percentage calculated excluding “not applicable” responses.

**Table 2.** Responses rated as “extremely important” or “very important” in determining progression of graded responsibility

Domain of practice	Post-graduate year level	Completion of certain rotation	Clinical Competency Committee recommendations	Faculty evaluations	Observation of having performed task previously	Simulation	Milestone assessment
Intubating trauma patients	38 (80.9)	25 (53.2)	10 (21.3)	7 (14.9)	25 (53.2)	9 (19.1)	12 (25.5)
Managing critically ill trauma patients	47 (94.0)	23 (46.9)	17 (34.0)	15 (30.6)	15 (30.6)	9 (18.0)	11 (22.4)
Managing critically ill medical patients	22 (88.0)	9 (36.0)	7 (28.0)	7 (28.0)	7 (28.0)	4 (16.0)	2 (8.0)
Acting as physician-in-triage	19 (100.0)	1 (5.3)	6 (31.6)	5 (26.3)	2 (10.5)	2 (10.5)	1 (5.3)
Supervising medical students	52 (92.9)	5 (9.1)	17 (30.9)	17 (30.9)	10 (18.2)	1 (1.8)	6 (10.9)
Supervising junior residents	36 (100.0)	4 (11.4)	20 (55.6)	15 (41.7)	11 (31.4)	4 (11.4)	5 (14.3)
Moonlighting	77 (100.0)	14 (18.4)	55 (71.4)	45 (58.4)	25 (32.9)	10 (13.2)	36 (47.4)

Values are presented as number (%). Response choices: not at all important, a little important, somewhat important, very important, and extremely important.

## Footnotes

**Data availability** Data files are available from Harvard Dataverse: https://doi.org/10.7910/DVN/CE74U6 Dataset 1. Data file and data dictionary.

## Supplementary materials

Data files are available from Harvard Dataverse: https://doi.org/10.7910/DVN/CE74U6

Supplement 1. Screen shot of web-based survey created to assess how the Accreditation Council for Graduate Medical Education (ACGME)-accredited emergency medicine residency programs implement graded responsibility among trainees across multiple domains of practice.

Supplement 2. The audio recording of the abstract.
