# Supplementary material for: Use of graded responsibility and common entrustment considerations among United States emergency medicine residency programs
Source: J Educ Eval Health Prof. 2020 Apr 20;17:11. doi: 10.3352/jeehp.2020.17.11 (PMC7225606; doi:10.3352/jeehp.2020.17.11)

## Slide 1
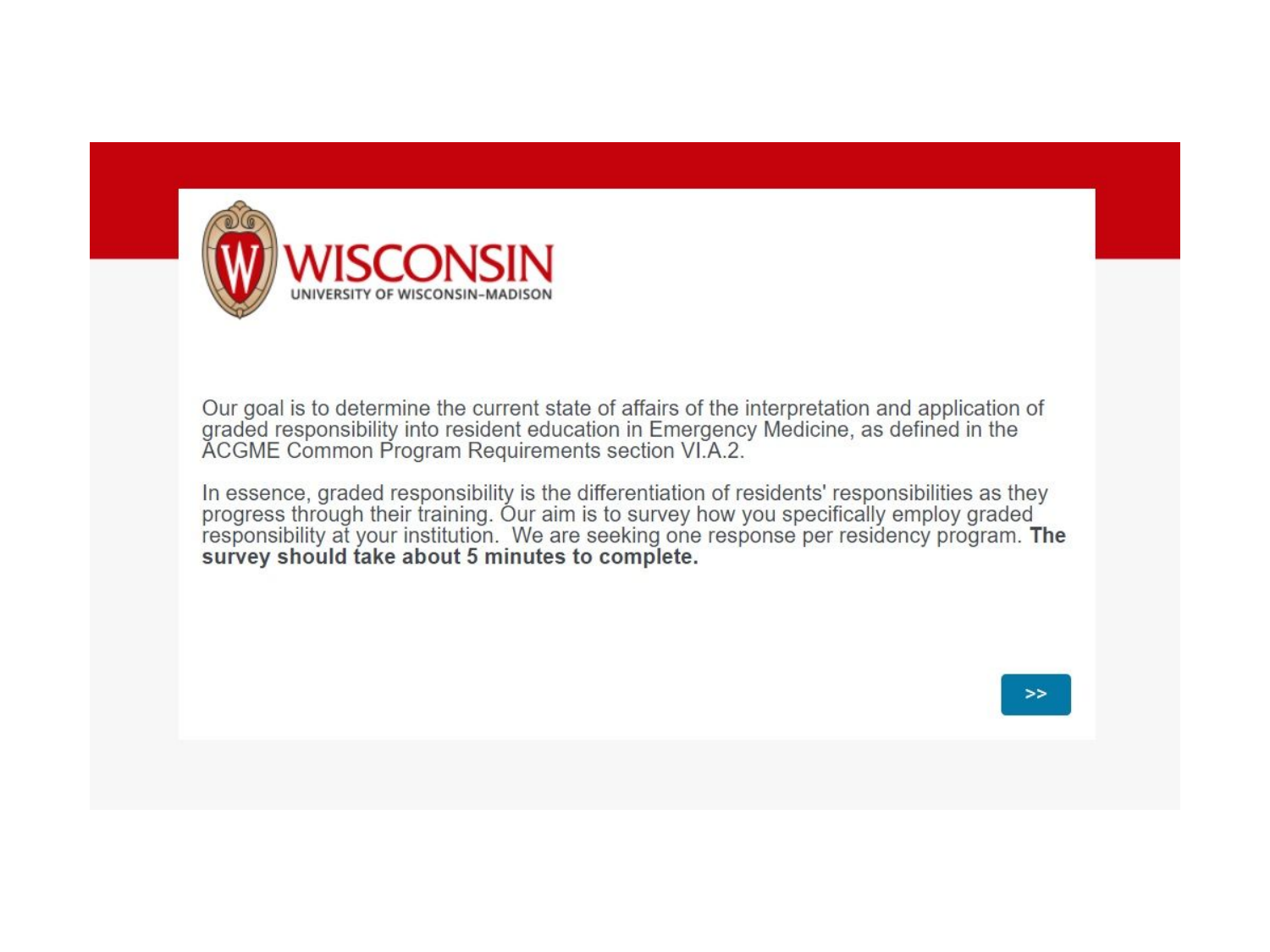

## Slide 2
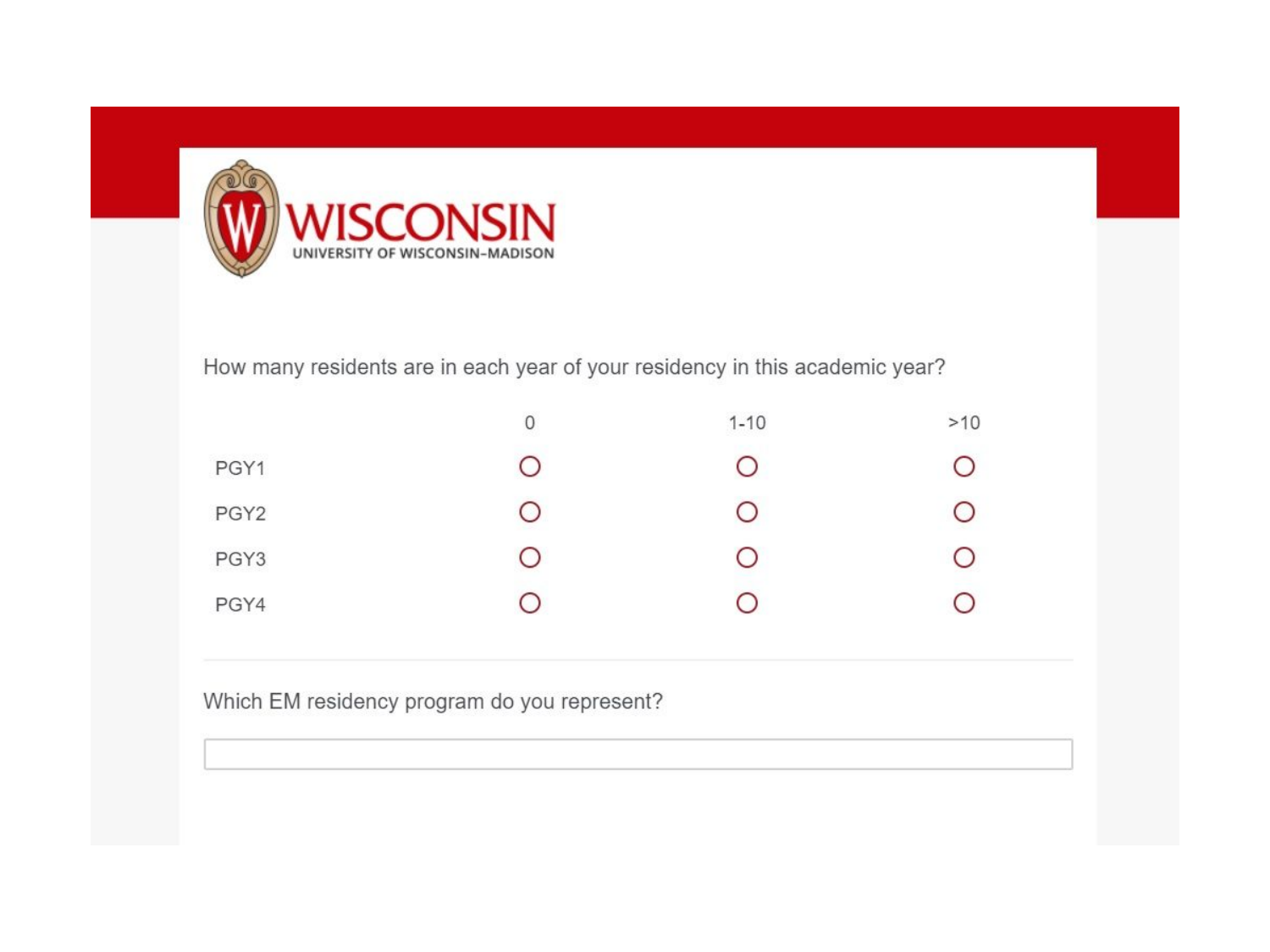

## Slide 3
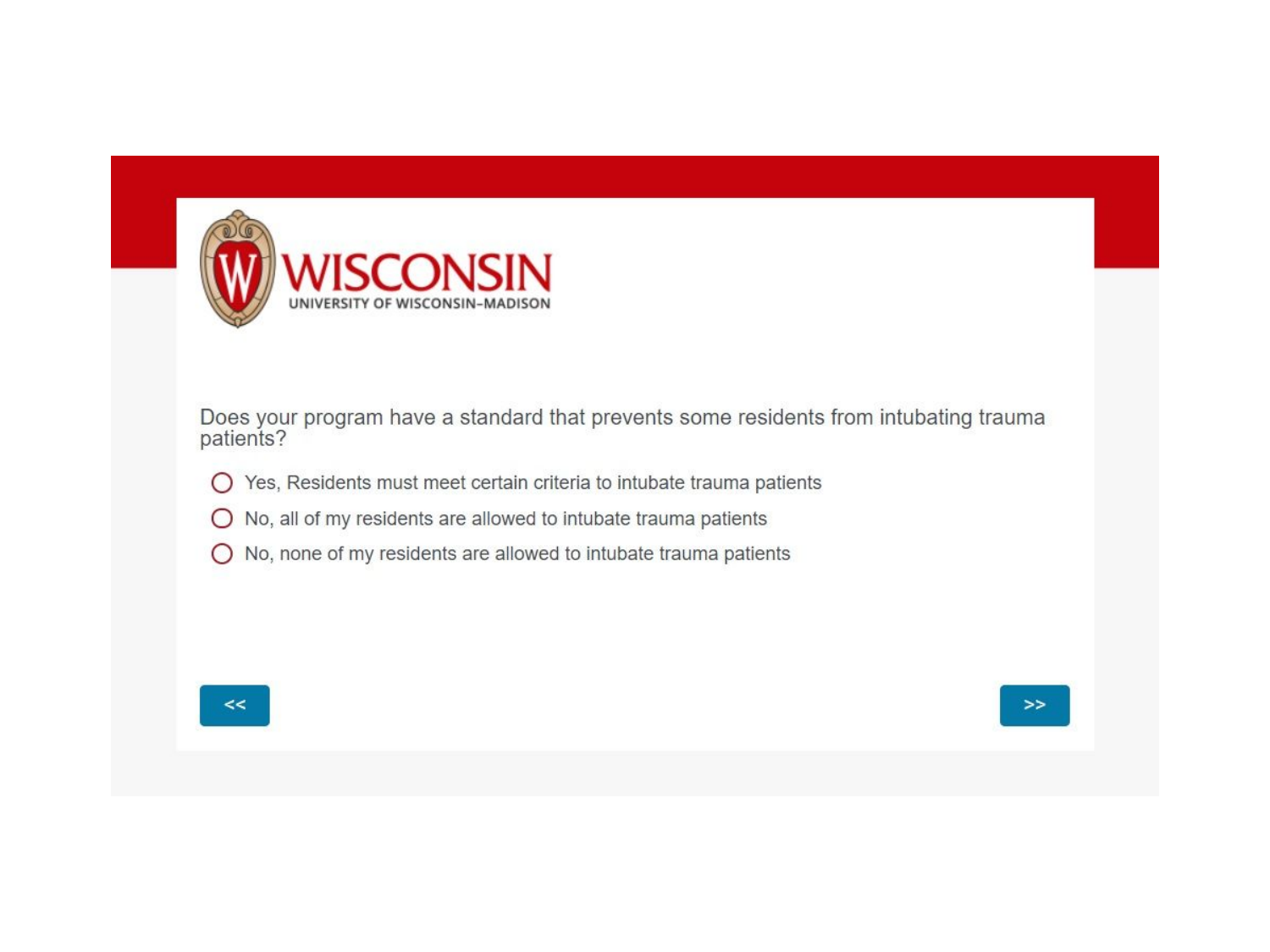

## Slide 4
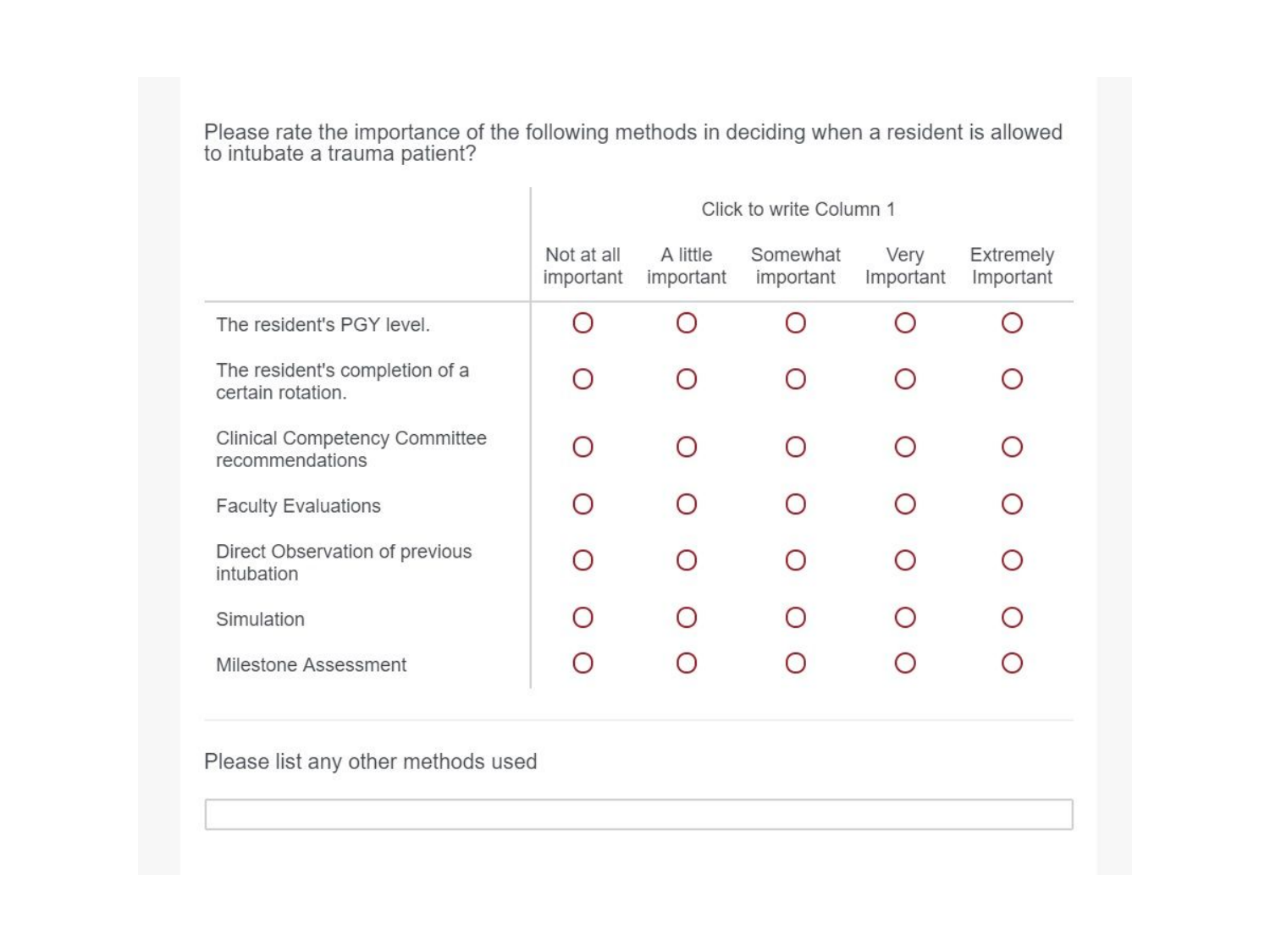

## Slide 5
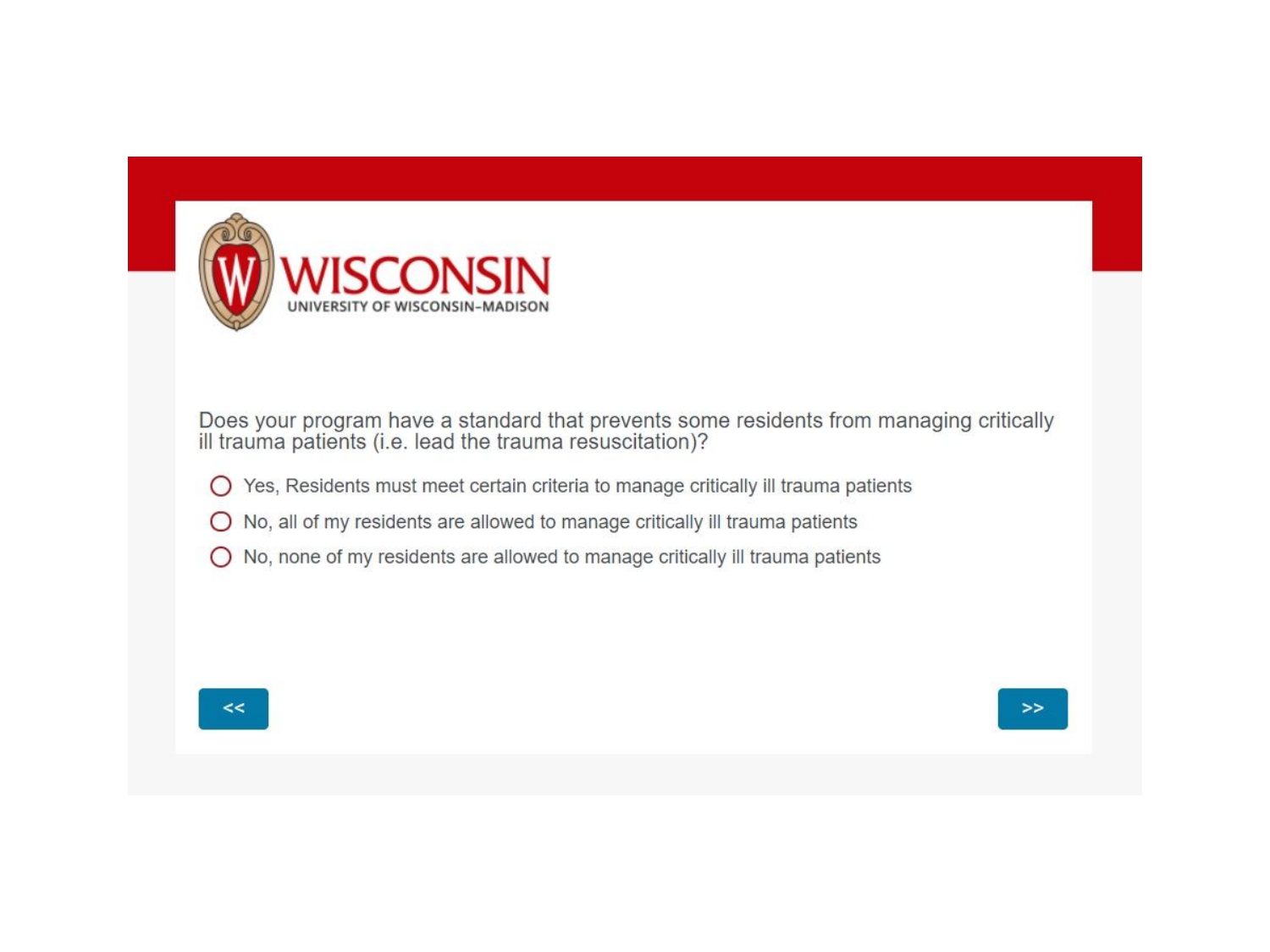

## Slide 6
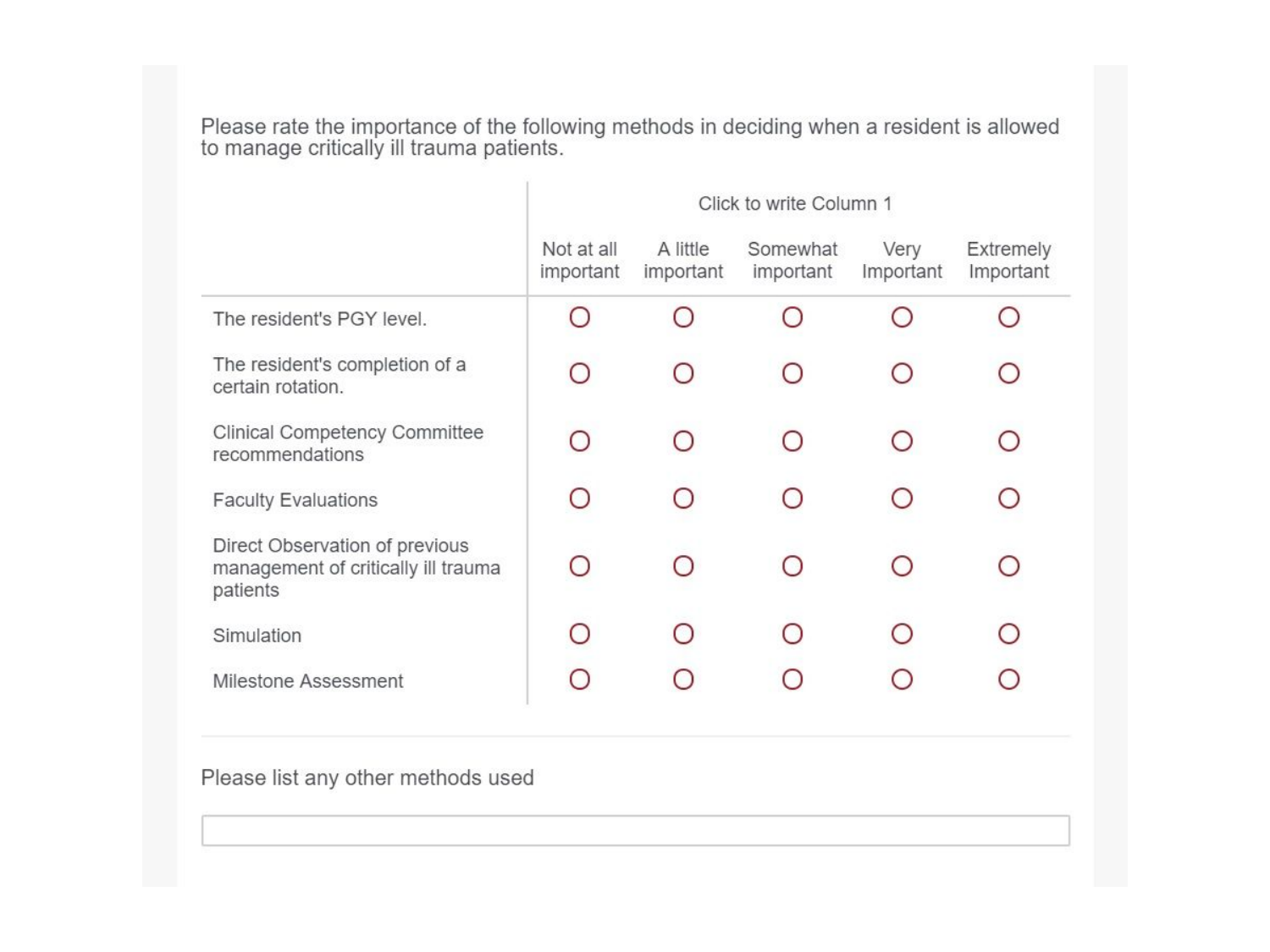

## Slide 7
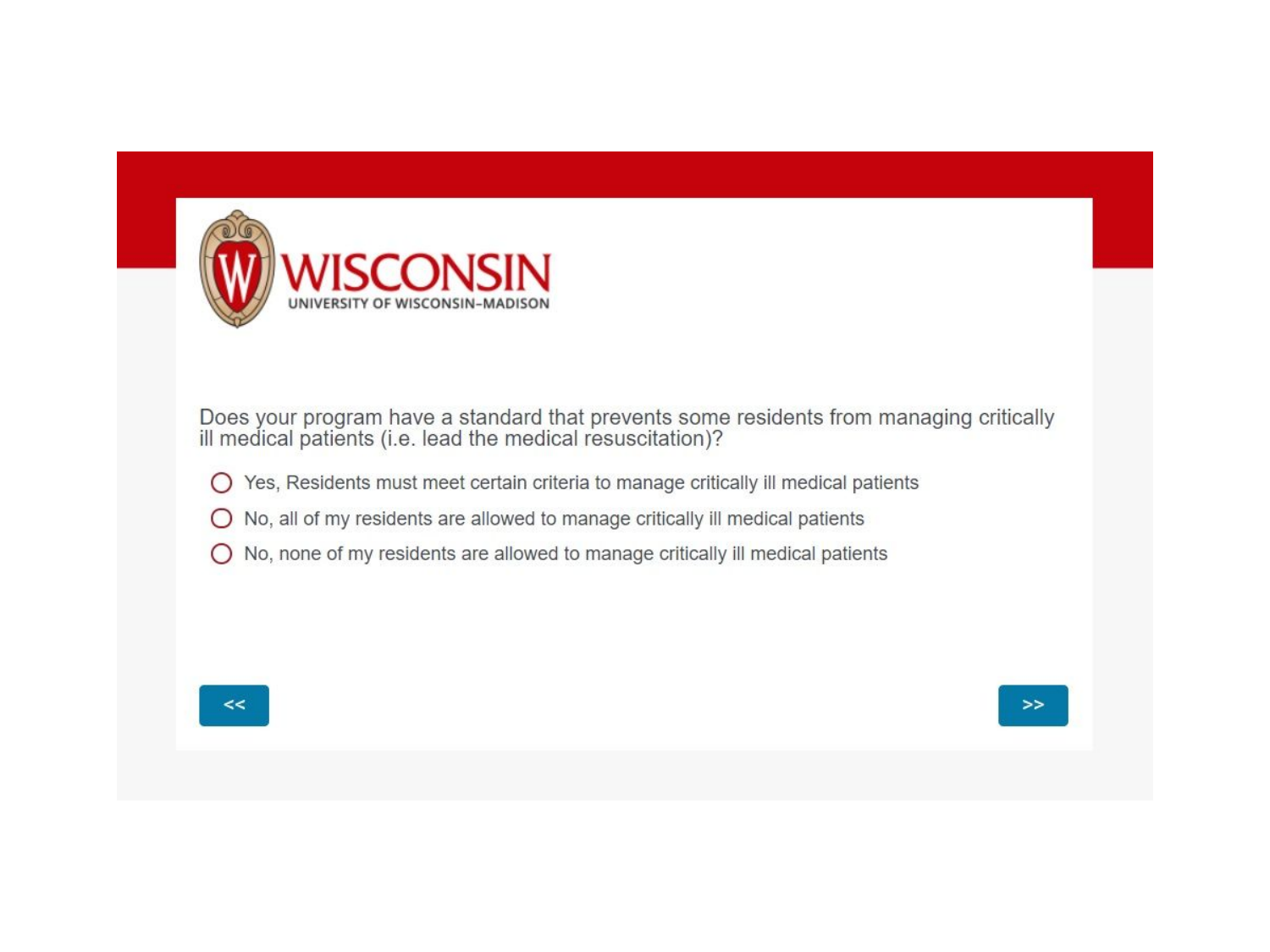

## Slide 8
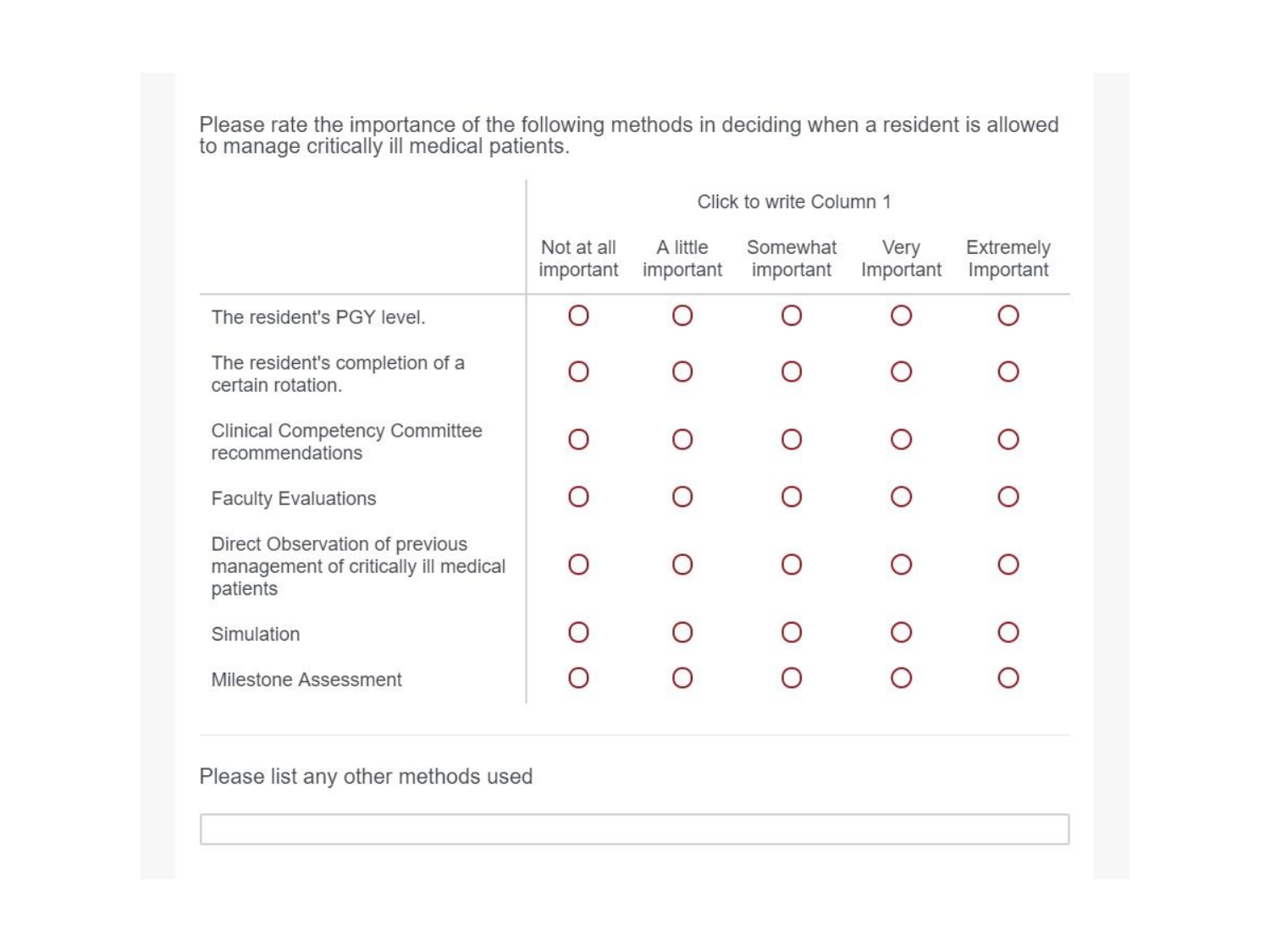

## Slide 9
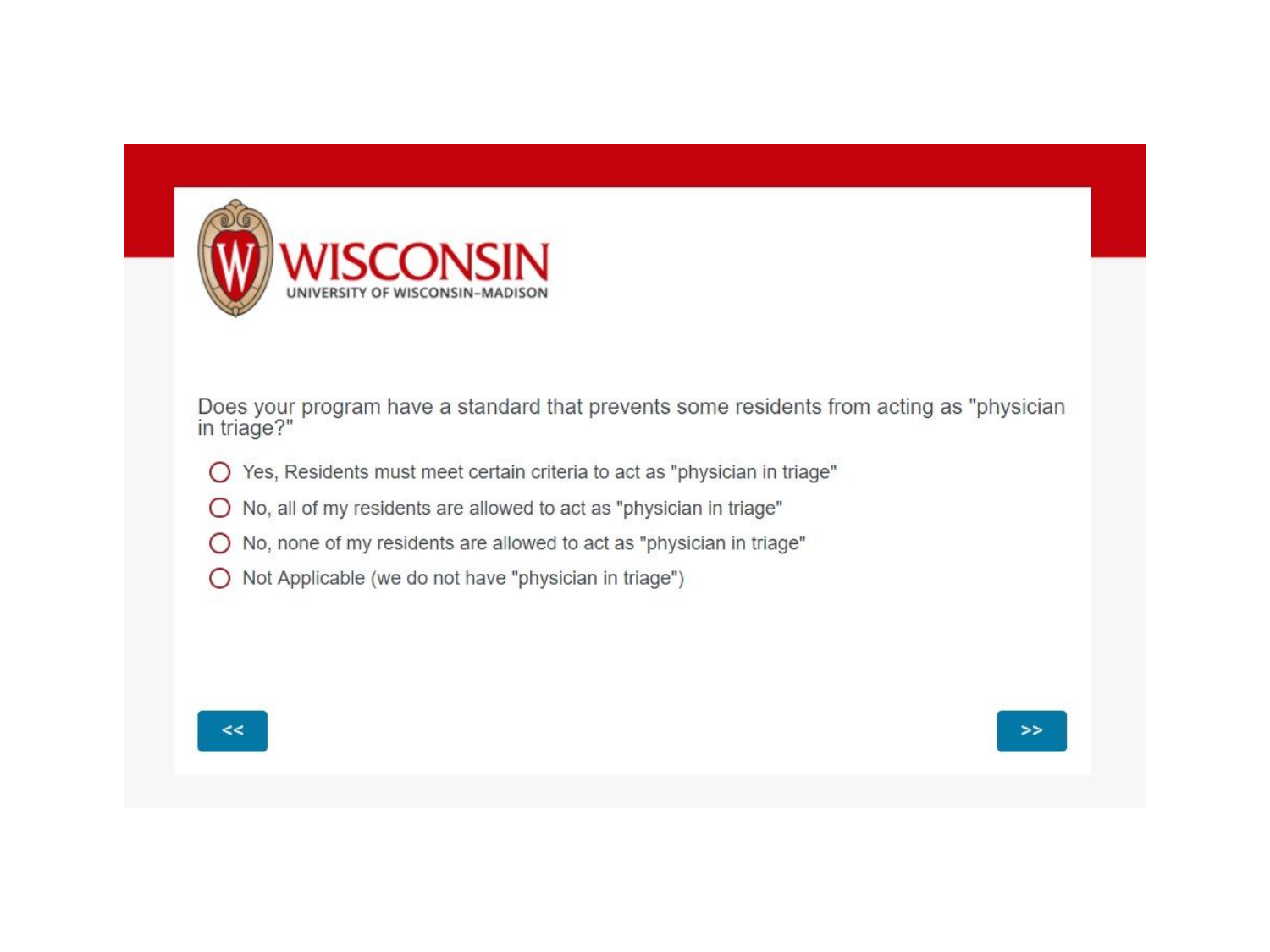

## Slide 10
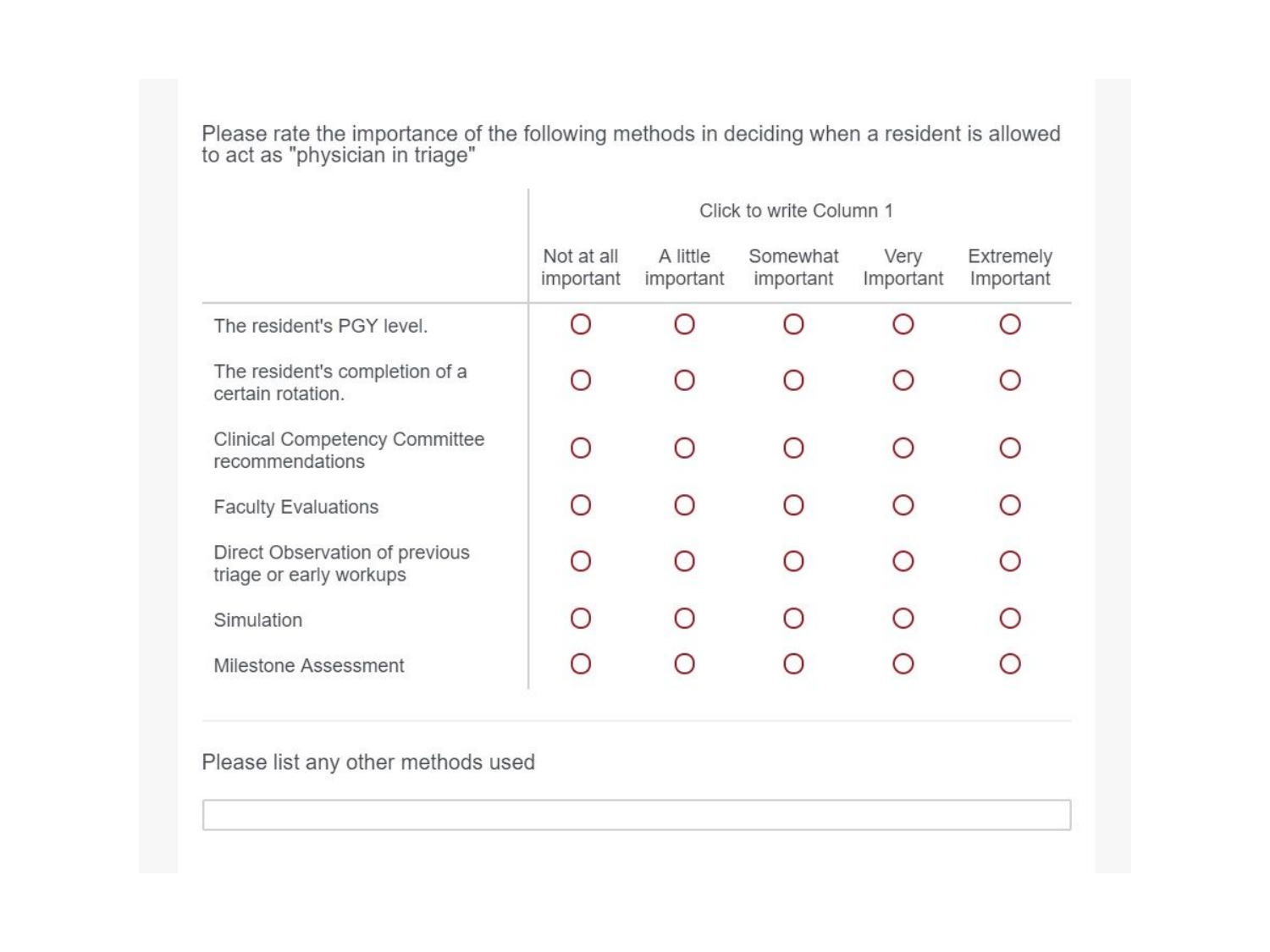

## Slide 11
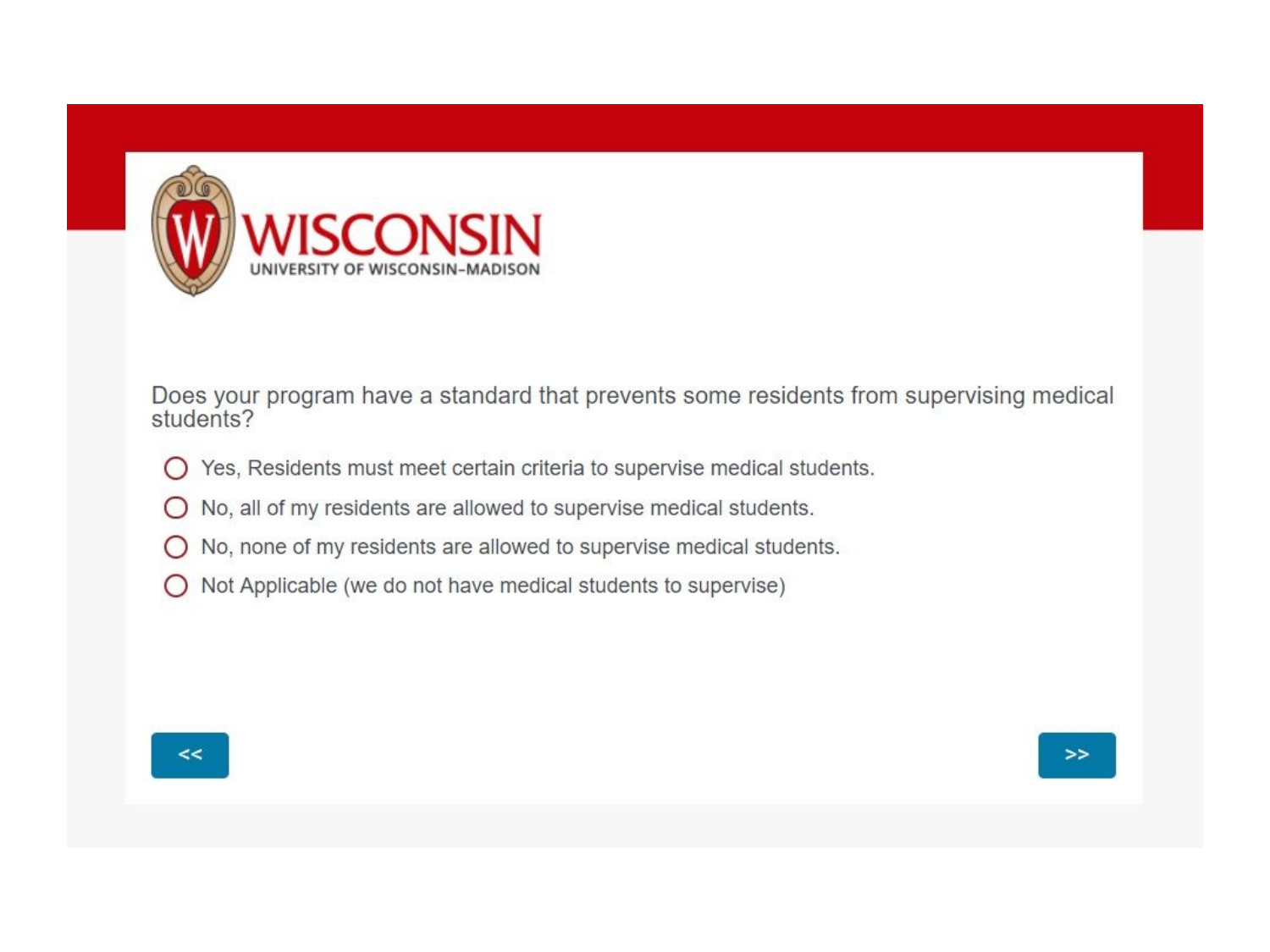

## Slide 12
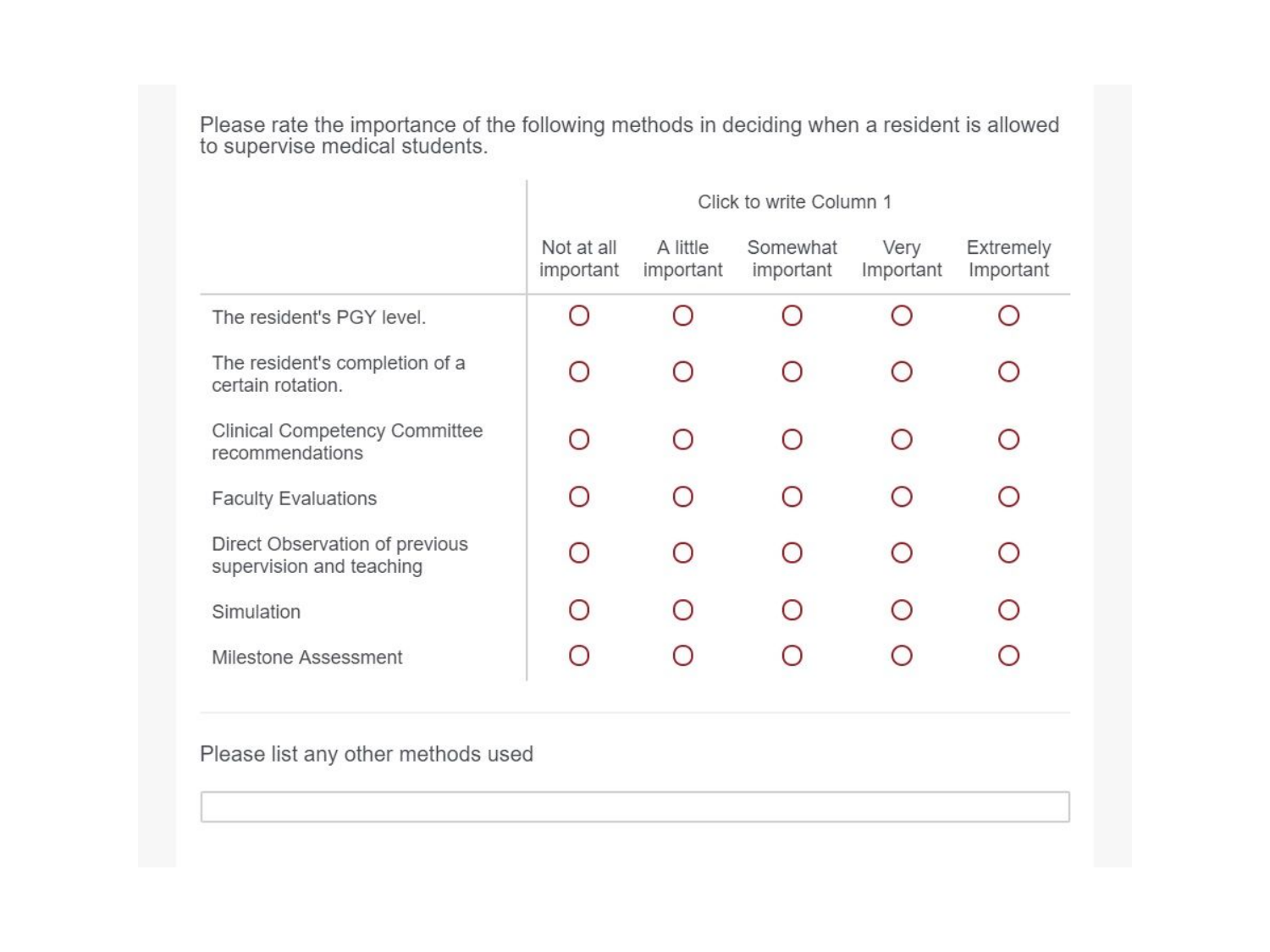

## Slide 13
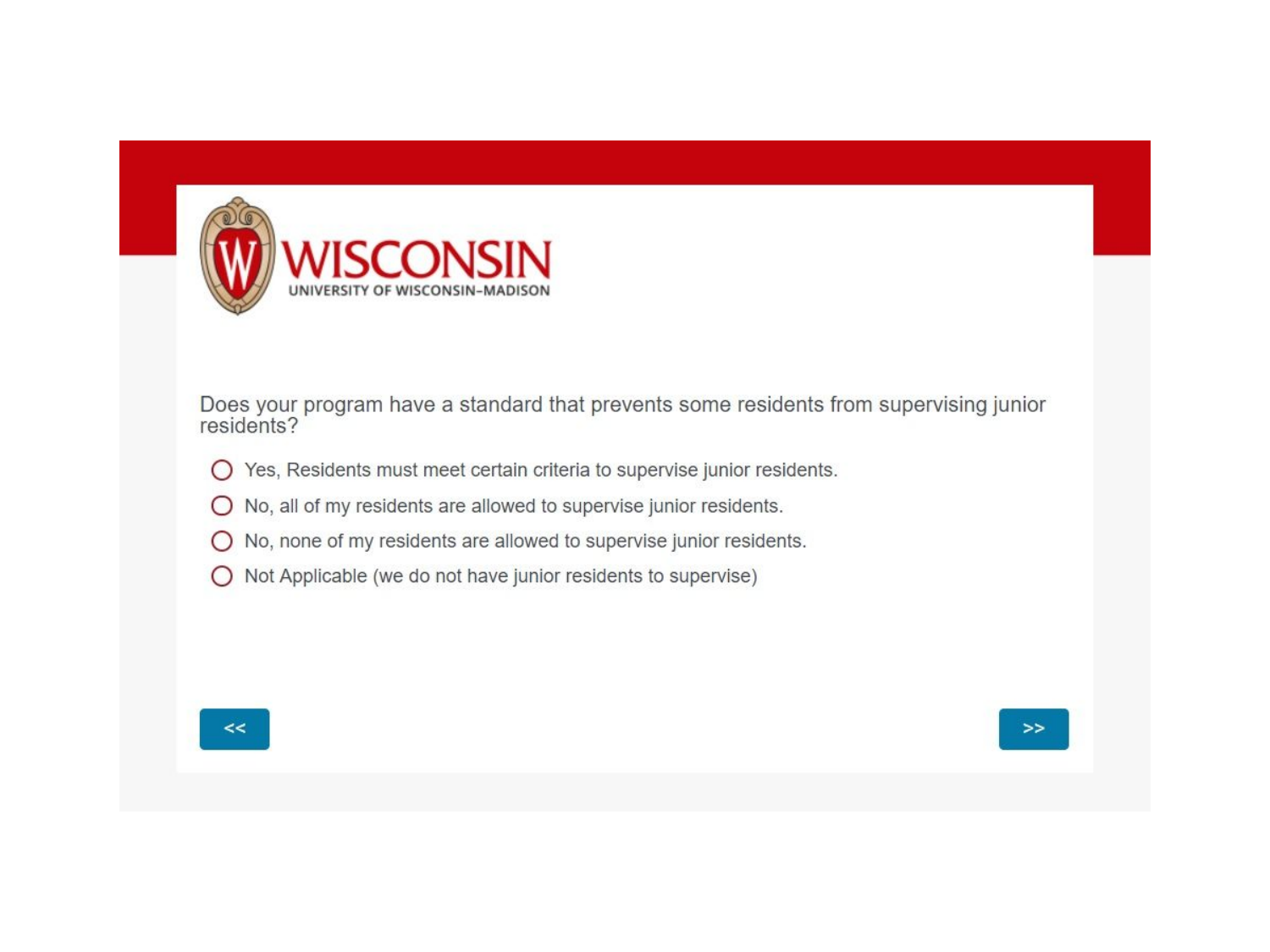

## Slide 14
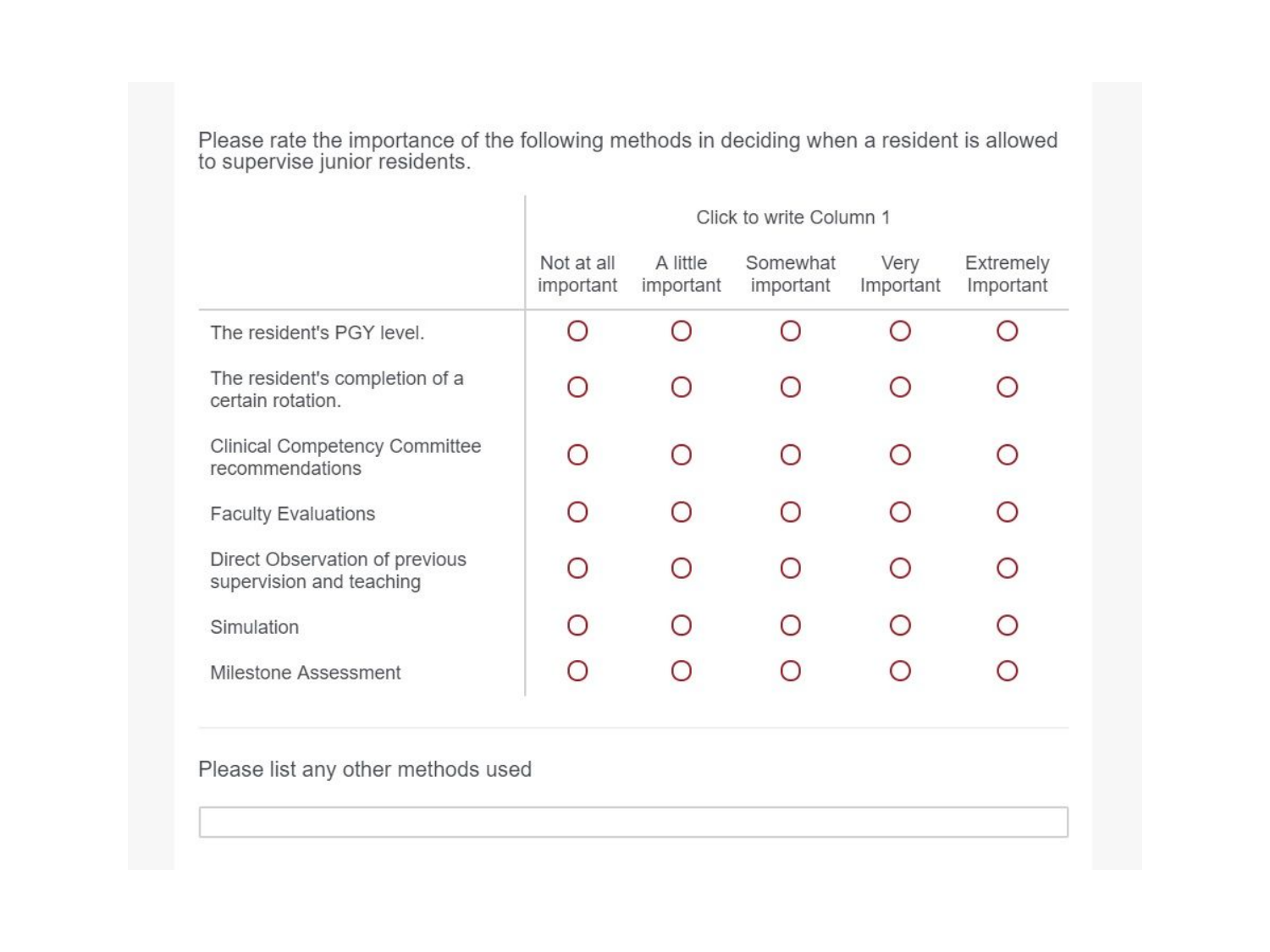

## Slide 15
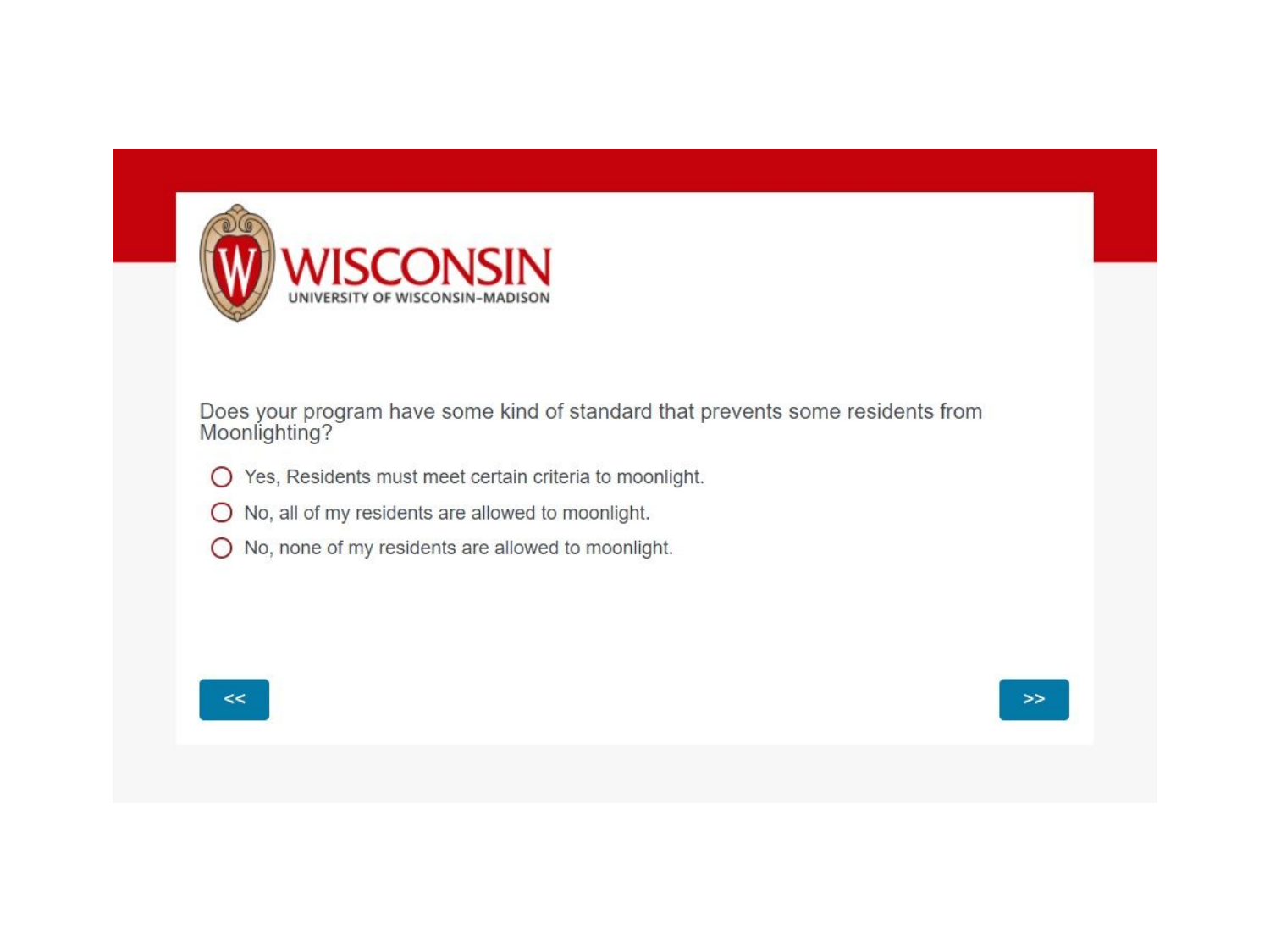

## Slide 16
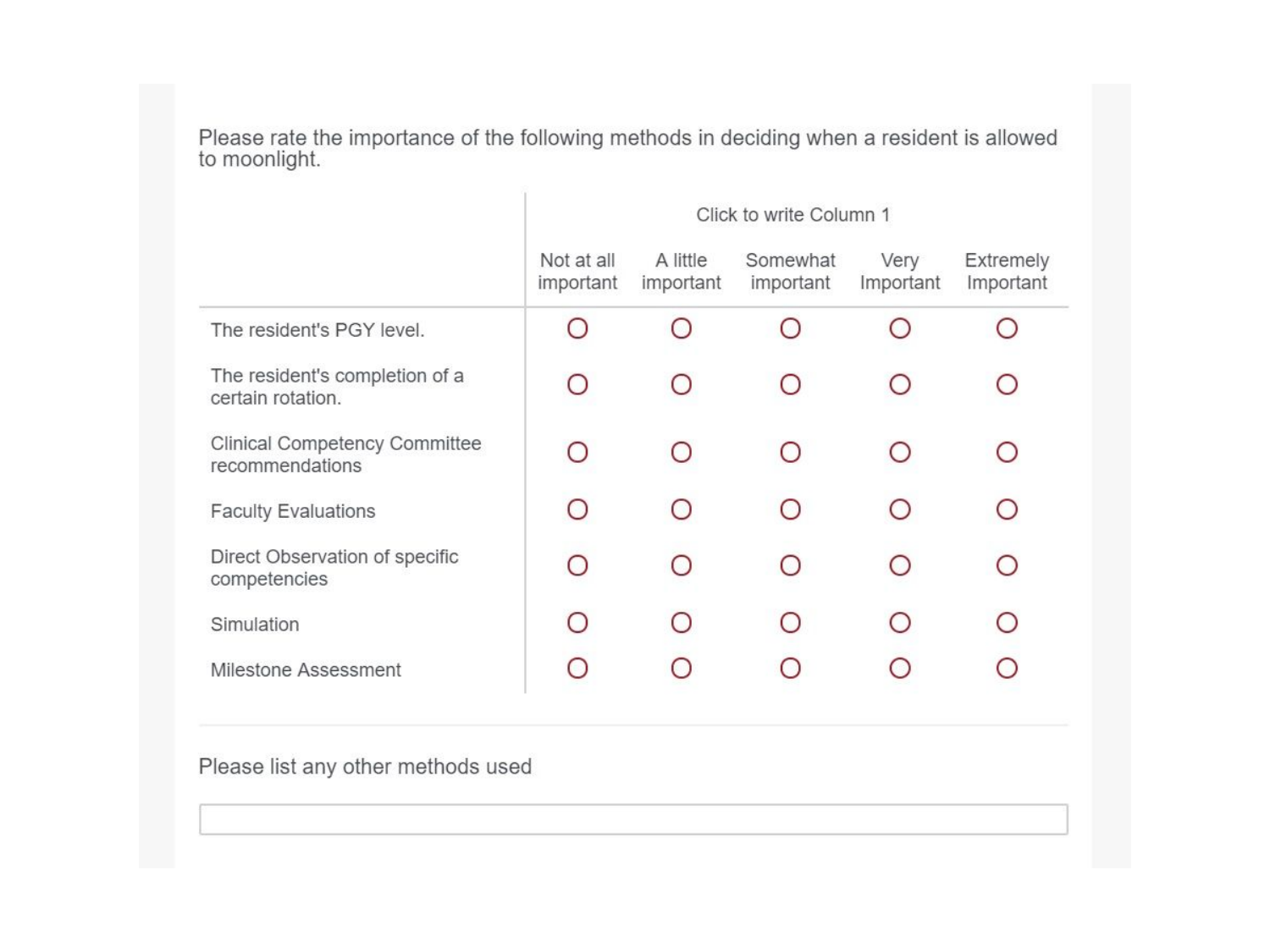

Supplement: Supplementary file 2 — Supplement 1. Screen shot of web-based survey created to assess how the Accreditation Council for Graduate Medical Education (ACGME)-accredited emergency medicine residency programs implement graded responsibility among trainees across multiple domains of practice. [file jeehp-17-11-suppl.pptx]
